# Non-polypoidal, synchronous mantle- cell lymphoma of small intestine: a rare case

**DOI:** 10.1186/1477-7819-8-69

**Published:** 2010-08-13

**Authors:** Nikolaos Sikalias, Konstantinos Alexiou, Maria Demonakou, Sylvia- Christina Mylona, Theodora Papadaki, Nikolaos Ekonomou

**Affiliations:** 1 1st Surgical Department, Sismanogleio General Hospital, (st. Sismanogliou 1st), Marousi - Athens (15126), Greece; 2 Pathology Department, Sismanogleio General Hospital, (st. Sismanogliou 1st), Marousi - Athens (15126), Greece; 3 Haematology Department, Evangelismos General Hospital, (Ave. Ypsilantou 45-47) Athens (10676) Greece

## Abstract

Herein is reported the case of a mantle cell lymphoma (MCL) with synchronous double intestinal location. A 74 - year old male presented with mild abdominal pain. CT scan imaging indicated invasion of lateral intestinal cavity by large mass formation. Exploratory laparotomy was performed and two solid extra-mural masses were isolated and excised. Histology revealed non- polypoid double synchronous lymphoma of mantle cell origin, an unusual presentation of the disease.

## Background

Mantle cell lymphoma (ICD-O code 9673/3) is a subtype in the wide category of B- cell lymphomas [1,2]. It is a specific type of Non- Hodgkin's lymphomas comprising 3% - 5% of all cases [1-3]. The histological derivation is from the "naïve" (functionally active but immunologically immature cells) CD5^+ ^B- cells residing in the primary follicles or in the mantle zones of secondary follicles [4]. A translocation between chromosomes 11; 14 takes place, leading to the juxtaposition of the cyclin D1 and the consequent over- expression of the CCND1 gene [5]. Mantle cell lymphoma (MCL) is composed of monomorphous small to medium sized lymphoid cells with irregular nuclei [1,2].

At the time of diagnosis most patients have signs of multiple lymphatic involvement, including spleen, red bone marrow, cervical lymph nodes, liver, and gastrointestinal tract, usually under a condition known as "multiple small intestine polyps" [6-8]. MCL cells may also invade the brain and spinal cord [6,8]. Most patients present with stage III to IV of the disease including lymphadenopathy, hepatosplenomegaly while over 50% include bone marrow involvement [1,2,6].

## Case report

A 74- year-old Caucasian male Greek patient, presented with mild abdominal pain and a history of recurrent gastrointestinal bleedings over the last few years.

At the time of admission at the hospital his general state was not indicative for an emergent situation. The patient complained for insisting abdominal discomfort, moderate flatulence and anorexia. Physical examination revealed abdominal distension with flat sounds at percussion, moderately decreased intestinal sounds, without signs of localised sensitivity or peritoneal irritation.

Blood tests demonstrated normal WBC count (7.500 cells/mm^3^) with inverted cellular type (Neutrophils: 77,3%, Lymphocytes: 12,5%), moderate decrease of Hematocrit and Hemoglobin (40% and 12,6 g/dl respectively) whereas Platelet count was noticeably elevated at 662.000 cells/mm^3^. Biochemistry was within normal levels, as well as coagulation time exams, except for elevated CRP count (at 45 mg/L) and decreased Albumin/Globulin ratio. Tumour marker CA 125 was also increased (600 U/ml - normal value < 35 in our lab).

**Figure 1:**
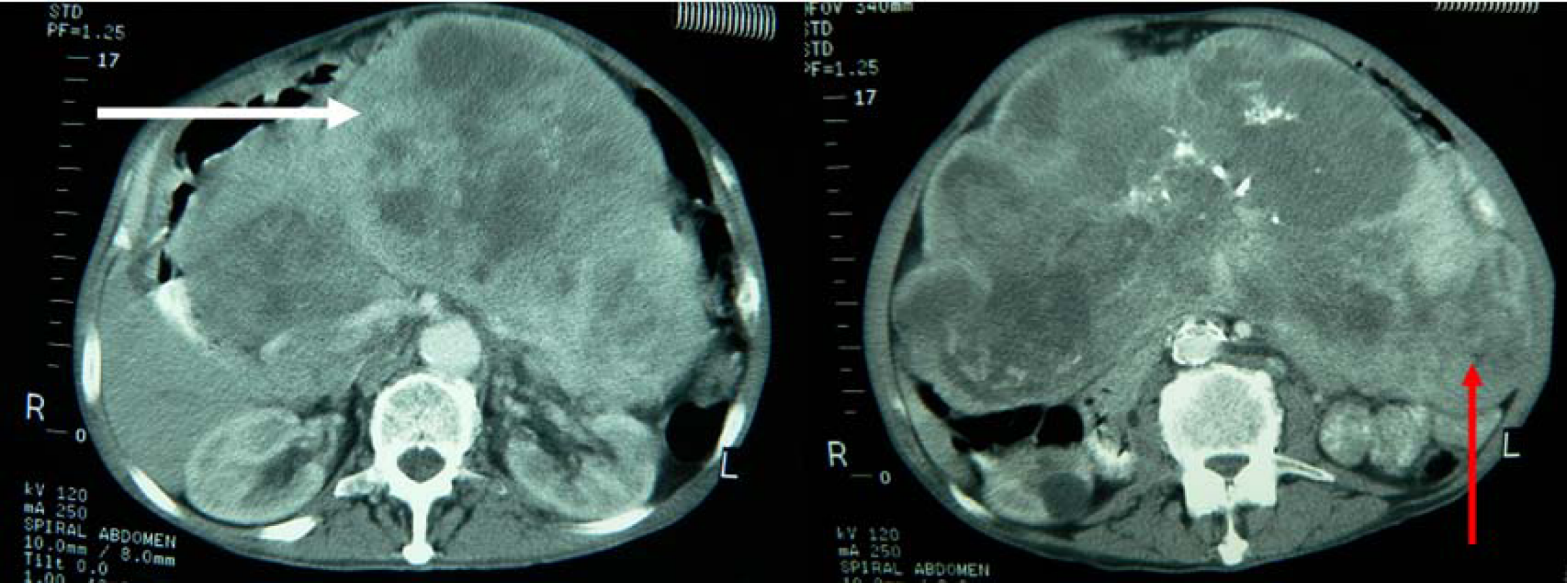
**Preoperative spiral abdominal Computed Tomography images revealing mass formations (white arrow pointing to the large tumor and the red arrow pointing to the small tumor)**.

An emergency abdominal CT scan revealed a large solid mass invading the left lateral area of the abdominal cavity and distended small bowel helixes (figure 1). There was also indication of partial intestinal obstruction, at the level of sigmoid colon, with imaging of hydroaeric levels and decreased transmission of the contrast agent. Gastroscopy and colonoscopy were also performed, both without confirmation of large bowel and sigmoid colon intraluminal obstruction.

**Figure 2:**
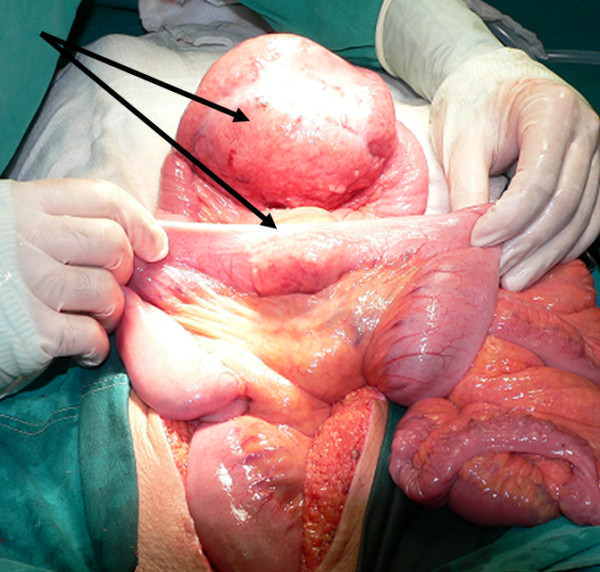
**Macroscopic aspect of tumoral formations intraoperativelly, before their excision (arrows pointing to the tumors)**.

**Figure 3:**
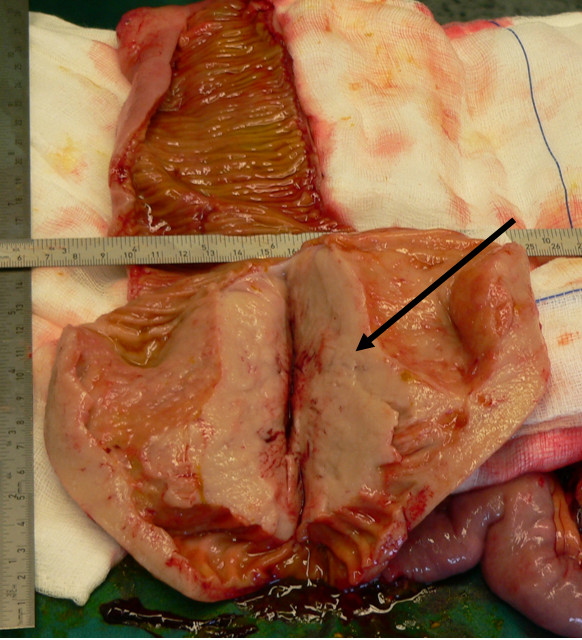
**Cut section of excised specimen intra operatively**. The main tumor located in ileum (13-14 cm length), which infiltrates transmurrally the intestinal wall (arrow pointing to the tumor).

**Figure 4:**
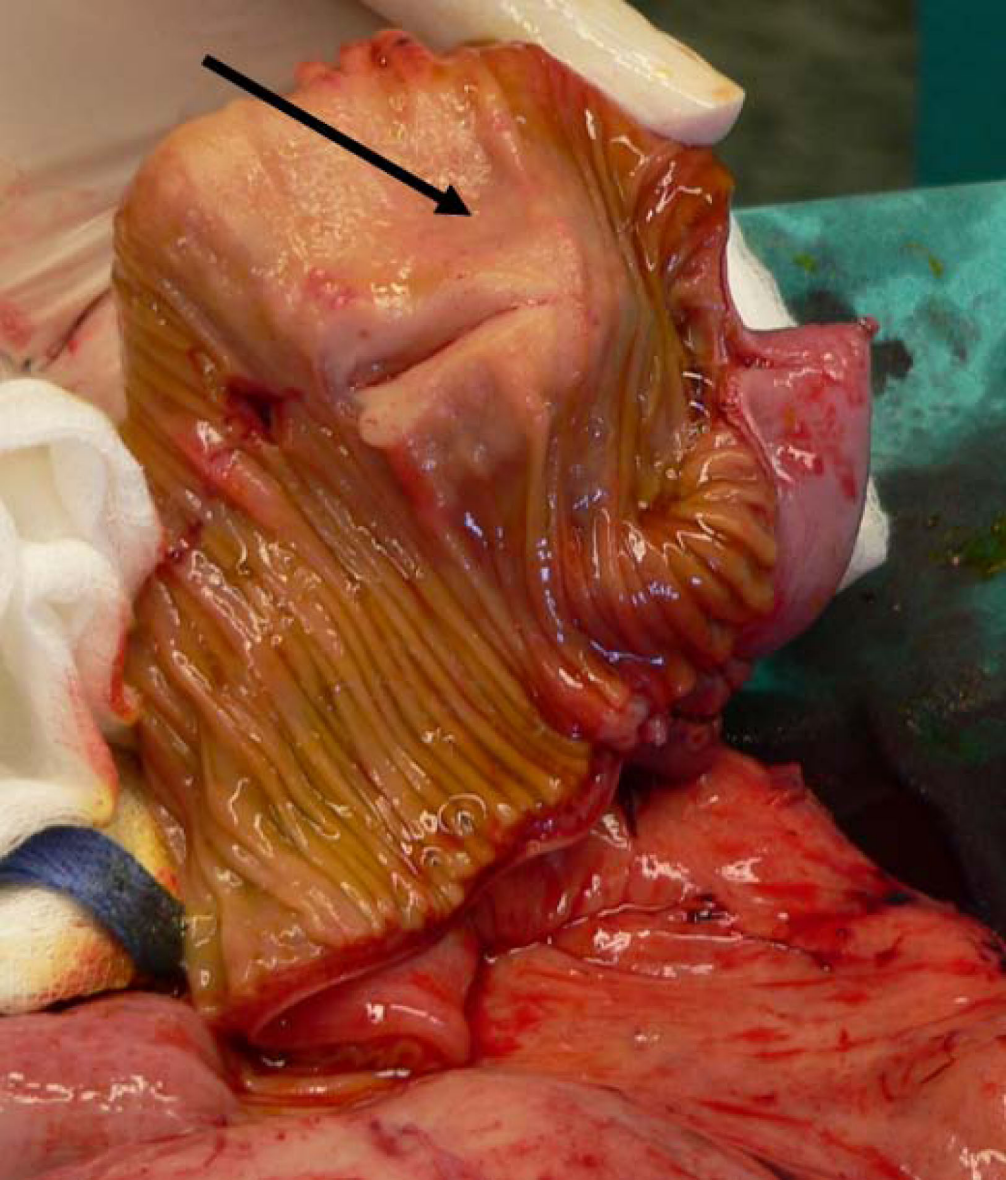
**Cut section of the second tumor also infiltrating the intestinal wall (arrow pointing to the tumor)**.

Exploratory laparotomy was performed through a vertical midline incision. A large extraluminal mass of approximate dimension 13 cm in diameter was detected at the level of ileum. Another smaller mass of 4-5 cm was revealed at 20 cm distance from the first finding, presenting similar macroscopic aspect. Both masses were solid in consistency, elastic and whitish in colour, extending transmurally through bowel walls (figures 2, 3, 4). No signs of abdominal obstruction, or distant implantations to other abdominal organs (liver, omentum) were confirmed.

A wide enterectomy of almost 37 cm was performed, with excision of small bowel, mainly ileum, followed by gastrointestinal anastomosis. Frozen section biopsy of the excised specimen was positive for malignancy. A large number of mesenteric lymph nodes were also included in the final biopsy material.

**Figure 5:**
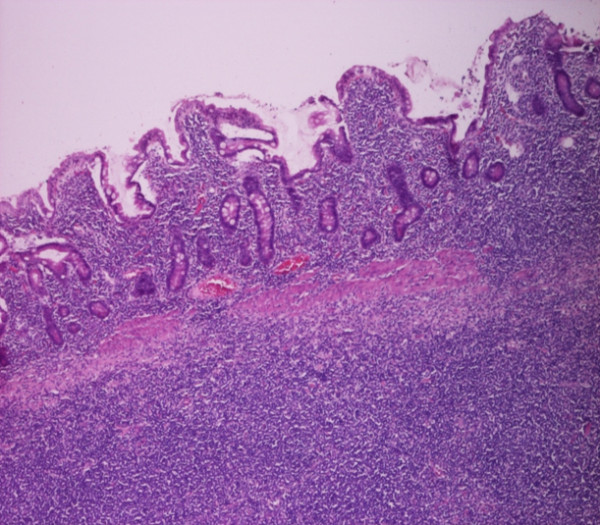
**Cytological appearance (HEX10):Intestinal Lyphoma Infiltrating mucosa and submucosa of ileum**.

**Figure 6:**
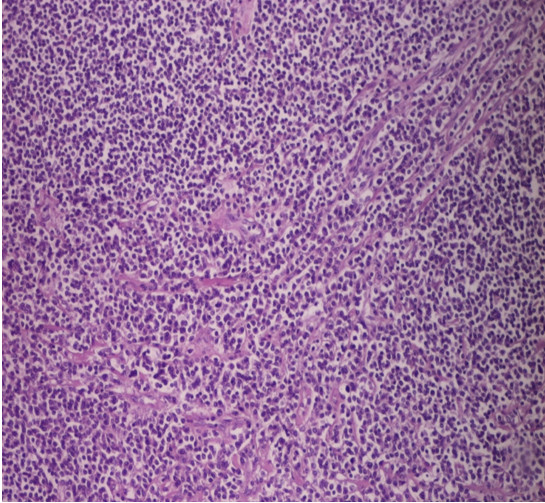
**Cytological appearance (HEX40): Neoplastic Cells occupie small intestinal mucosa**.

**Figure 7:**
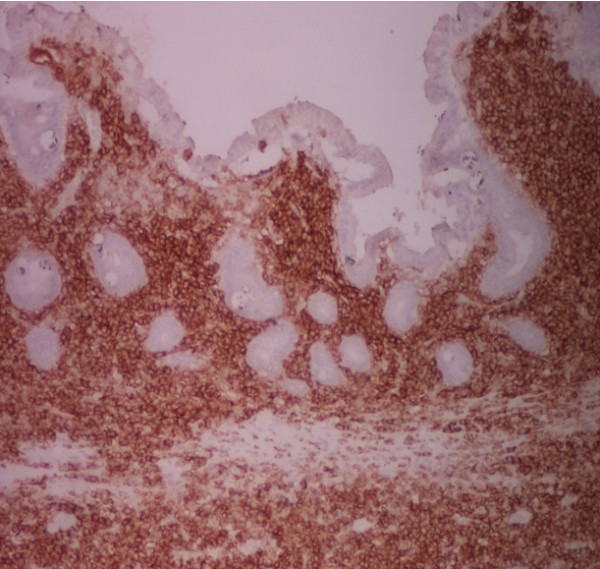
**Immuno histochemical stain with APAP/CD20 × 40: Neoplastic cells with strong positivitivy with a PanB marker**.

**Figure 8:**
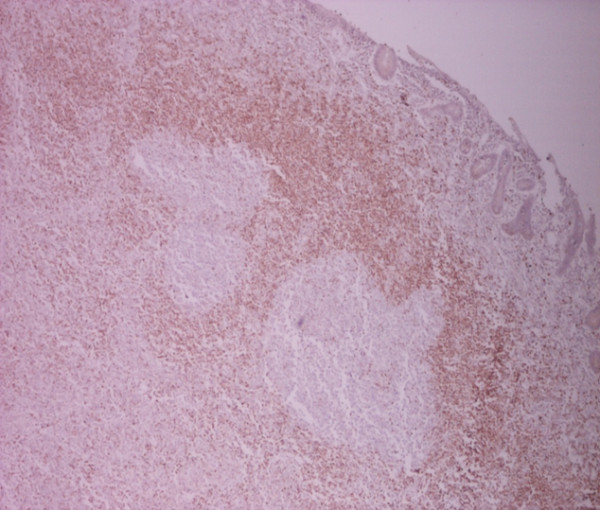
**Immuno histochemical stain with APAP/BCL2 × 25: Lymphoid follicles with expansion of mantle zone**.

Histology revealed diffuse, full- length, non- specific nodular infiltration of small bowel walls reaching the level of serosa, for both tumoral formations (figures 5, 6, 7, 8). Identification of cellular populations and immunohistological evaluation revealed a biphasic pattern with lymphocytic collections formed particularly by small lymphocytes mixed with a small population of immunoblasts, positive for Cyclin D1, CD5, CD20, CD35, CD79, CIgG(κ), and negative for CD3, CD10, CD21, CD23, and CD43. Limited cellular multiplication rate was noted. Plasmocyte populations were also noticed (positive for CD138, MUM-1, CIgG(κ) and negative for CD3 and CD20). Findings were significant for non- Hodgkin lymphoma of B-cell origin with low malignant potential. The tumoral origin is in the marginal cell zone demonstrating plasmocytic differentiation and positive CIgG(k) clonal functions. Furthermore, involvement of 14 mesenteric lymph nodes was confirmed.

The patient's postoperative course was uncomplicated, with immediate mobilisation and normal intestinal functionality. He was discharged from hospital 10 days later in a general good status and after an interval of 22 days he initiated adjuvant chemotherapy (CHOP regiment) administered in a single day every 3 weeks for 6 cycles. In addition to chemotherapy the patient received treatment with monoclonal antibody Rituximab which acts against CD20, a special molecule on the surface of B-cell Non-Hodgkin Lymphomas.

A complete physical and imaging evaluation 21 months later revealed complete recovery and no signs of remaining disease.

## Discussion

Mantle cell lymphoma is an entity of B- cell malignancies, belonging in the non- Hodgkin lymphomas [1,2,6]. Intestinal lymphoma is the most usual presentation of extranodal MCL, presenting under the form of multiple lymphomatous polyposis (MLP). Small intestine (duodenum, jejunum and ileum) is reported to be involved in 15% - 30% of GI lymphomas [9,10].

Prognosis for intestinal malignant lymphoma is poor due to its accelerated proliferation and the non- specific clinical presentation of the disease. Most of the patients have advanced stages of the disease at the time of diagnosis, being also delayed by the difficulty in direct visualization of small intestine. Patients with obstructive tumour masses require surgical resection.

Chemotherapy is the treatment of choice, including regimens with (1) Cyclophosphamide, Hydroxydaunorubicin, Oncovin, Prednisone or Prednisolone (CHOP), (2) Cyclophosphamide, Vincristine, and Prednisone (COP), and (3) Doxorubicin, Tteniposide, Cyclophosphamide, and Prednisolone (AVmCP). Reports after application of AVmCP regimen, reach response rate of 80%, with a 5- year survival rate of 59%. Less aggressive regimens, such as COP, appear to be less effective, with a response rate of 30% and a mortality rate of 100% 3 years after treatment [4,9].

The case of extra- luminal augmentation of the tumour is extremely seldom and double synchronous presentation of such masses even rarer. Few cases are reported of synchronous colonic tubular presentations [10,11]. In non intestinal locations, MCL of the pleura and synchronous presentation of pulmonary adenocarcinoma may present [2] and there is also case of metastatic MCL presenting as a prolapsed vaginal mass [12].

## Conclusions

Non-polypoid lymphomas of the small bowel can be a rare place of occurrence for mantle cell lymphomas. In such cases of lymphomas presenting as small bowel individual tumors, with single or multiple location, the treatment of choice is surgical resection with appropriate anastomotic procedures. Following surgery, additional treatment with chemotherapy is also proposed, according to the classification of malignancy.

## Abbreviations

MCL: mantle cell lymphoma; CT: computed tomography; WBC: white blood cells; CRP: c-reactive protein; MLP: multiple lymphomatous polyposis;

## Consent

Written informed consent was obtained from the patient for publication of this case report and accompanying images. A copy of the written consent is available for review by the journal's Editor-in-Chief.

## Competing interests

The authors declare that they have no competing interests.

## Authors' contributions

SN, AK, EN have had an equally substantial contribution to the clinical diagnosis, surgical management and post-op follow-up of the patient. DM and PT analysed the specimen and confirmed the diagnosis, MSC and SN drafted the manuscript. SN and EN are guarantors of the paper. All authors read and approved the final manuscript.
